# Effectiveness of non-pharmacological interventions for treating post-stroke depression: Study protocol of an umbrella review of systematic reviews and meta-analyses

**DOI:** 10.1097/MD.0000000000028370

**Published:** 2021-12-23

**Authors:** Chengcheng Zhang, Lianyi He, Zhendong Li, Hangjian Qiu, Xiaoqian Wang, Yuejuan Zhang

**Affiliations:** a Hunan University of Chinese Medicine, Changsha, Hunan, China; b The First Affiliated Hospital of Hunan University of Chinese Medicine, Changsha, Hunan, China.

**Keywords:** non-pharmacological treatment, post-stroke depression, protocol, study, systematic reviews

## Abstract

**Background:**

Many systematic reviews and meta-analyses have evaluated the effectiveness of non-pharmacological therapies to improve symptoms of post-stroke depression (PSD) and reduce disability and mortality in patients with PSD. However, no research has appraised the credibility of the evidence. This study aims to summarize and evaluate the current evidence for non-pharmacological treatment of PSD and to seek effective treatment with reference to reliable evidence.

**Methods:**

We searched the electronic databases EMBASE, MEDLINE, Cochrane Central, PubMed, PROSPERO, Web of Science, and CINAHL. We will search articles from the above database for all published meta-analyses to December 2021 to evaluate the effect of non-pharmacological treatment of PSD. Two reviewers will extract the general characteristics of the included articles, as well as participants, interventions, outcome measures, and conclusions. The quality evaluation of each systematic review will be conducted with reference to the AMSTAR 2 tool. The effect size of each review will be recalculated using either a fixed-effects or a random-effects model. Cochrane's Q test and *I*^2^ statistics will be used to evaluate the heterogeneity between studies. To determine whether a systematic review had small study effects, we will use the Egger test. We expect to extract valid evidence and classify it from strong to weak.

**Results:**

The findings of this umbrella review will provide effective evidence for the non-pharmacological treatment of PSD.

**Conclusion:**

Our research conclusion will provide clinical staff and PSD patients with appropriate treatment recommendations.

**Ethics and dissemination:**

As the data were obtained from published materials, there is no need for ethical approval for this umbrella review. The findings of this umbrella review will be published in a peer-reviewed journal.

**INPLASY registration number:**

INPLASY2021100083.

**Strengths and limitations of this study:**

- This umbrella review will comprehensively evaluate the credibility of the evidence for the nonpharmacological treatment of PSD.
- This review will use quantitative analysis to assess heterogeneity between studies, small study effects, and excessive significant bias. We will distinguished the credibility level of each metaanalysis according to the corresponding criteria.
- There are many non-pharmacological treatments for PSD, and we may not be able to include all studies that meet predetermined requirements.

## 1 Introduction

Stroke is an acute cerebrovascular disease that causes brain tissue damage due to sudden rupture or blockage of blood vessels in the brain, which prevents blood from flowing into the brain.^[1]^ As a major public health problem, stroke is one of the most common causes of disability and death.^[2]^ Post-stroke depression (PSD) is a major complication of stroke, affecting approximately one-third of strokes in the recovery period. ^[3]^ In addition, depressive symptoms often appear in the first year after stroke, and patients with prior depression or severe stroke have a higher incidence of depression.^[4,5]^ The pathogenesis of PSD remains unclear, and it may include neurobiological dysfunction and psychosocial factors.^[6,7]^ The symptoms of PSD mainly manifest as decreased interest, mental retardation, restlessness and pessimism, insomnia, early awakening, and general fatigue, which not only affects the mental health of patients, but also affects the rehabilitation and exercise of patients, reduces compliance with medication, and greatly increases the disability rate and mortality of stroke patients.^[8,9]^ PSD not only increases the suffering of patients, but also imposes a huge economic burden on the family and society.^[10]^

Currently, clinical therapies for PSD are still dominated by drugs. Frontline drugs for the treatment of PSD mainly include selective serotonin reuptake inhibitors (SSRIs), serotonin-norepinephrine reuptake inhibitors (SNRIs), and tricyclic antidepressants (TCAs). However, there is no definite evidence of a specific medicine for PSD.^[11]^ Pharmacological therapy can increase the risk of blurred vision, sexual dysfunction, urinary retention, tremors, severe insomnia, hypotension, and cerebral hemorrhage.^[12]^ In addition, the treatment of antidepressants may be limited by the potential risk of drug interactions.^[13]^ There are many non-pharmacological therapies for PSD that are safe, effective, and practical.^[14]^ There have been some randomized controlled trials (RCTs) to study the effect of non-pharmacological intervention on PSD, and researchers have produced relevant reviews on this basis. Many meta-analysis results have shown that non-pharmacological therapy is effective for PSD. Acupuncture seems to improve cognitive function and depressive symptoms in post-stroke patients.^[15]^ Exercise interventions have also been shown to be effective in patients with PSD.^[16]^ Cognitive behavioral therapy has a positive effect on improving depressive symptoms in PSD patients.^[17]^ Although reviews have shown the effectiveness of non-pharmacological therapies, the quality of studies is affected by inter-study heterogeneity and the risk of other biases, and the reliability remains to be confirmed.

An increasing number of RCTs on non-pharmacological treatments for PSD have been conducted over the years. Many studies have conducted meta-analyses on the clinical evidence of the treatments of PSD with non-pharmacological therapy, but the conclusions of meta-analyses are different, and the quality of methodology and the reliability of evidence remain unclear. On the basis of the above reasons, this study will adopt an umbrella review to systematically collect systematic reviews or meta-analyses of related non-pharmacological therapies for PSD, objectively evaluate the methodological bias, inter-study heterogeneity, reliability of conclusions, and to screen out the best evidence of non-pharmacological therapy to provide basis and suggestions for clinical research.

## 2 Methods and analysis

### 2.1 Study design

On the basis of the diversity of current treatments for PSD, there have been many systematic reviews and meta-analyses involving non-pharmacological treatment of PSD. We attempt to use an umbrella review to evaluate the trustworthiness and validity of the current evidence and determine reliable evidence without the risk of bias. Systematic reviews and meta-analyses of RCTs will be included by us, and reviews that have not quantitatively analyzed the original research will be excluded. We will select reviews that include or use non-pharmacological treatments alone to compare with placebo or conventional controls, and exclude traditional reviews, systematic reviews or meta-analyses in the planning stages, conference abstracts, non-quantitative synthetic meta-analyses, letters, and non-RCT meta-analyses to avoid bias in the final analysis. The protocol for this umbrella review is designed to match Preferred Reporting Items for Systematic Reviews and Meta-Analyses Protocols (PRISMA-P) guidelines.^[18]^ This study protocol has been registered in INPLASY (http://inplasy.com) with a unique ID of INPLASY2021100083.

### 2.2 Search strategy

We will conduct an in-depth search of systematic reviews and meta-analyses, including a review of non-pharmacological therapy as a monotherapy or as a supplement to drugs for the treatment of PSD. We consider searching the electronic databases EMBASE, MEDLINE, Cochrane Central, PubMed, PROSPERO, Web of Science, and CINAHL, and we will search articles from the above database for all published meta-analysis to December 2021. We did not consider language restrictions in the retrieved articles. To avoid omissions, we will conduct a literature search in the form of a combination of Medical Subject Headings (MeSH) terms and free text terms. These search terms will be combined to obtain a more comprehensive search articles and make corresponding adjustments according to the indexing strategies of different databases. PubMed's “similar literature” function was used to find more potential articles that met the inclusion criteria. In addition, references included in the study were tracked back to supplement the relevant reviews. The specific search strategy of the PubMed database is summarized in Table 1, and other online databases adopt a similar search strategy.

**Table 1:** Detailed search strategy in PubMed.

No.	Search Items
#1	“Stroke”[Mesh] OR “Cerebrovascular Accident”[Title/Abstract] OR “Brain Vascular Accident”[Title/Abstract] OR “Stroke, Cerebrovascular”[Title/Abstract] OR “Cerebrovascular Apoplexy”[Title/Abstract] OR “Acute Stroke”[Title/Abstract] OR “Cerebrovascular Accident, Acute”[Title/Abstract]
#2	“Depression”[Mesh] OR “Depressive Symptom”[Title/Abstract] OR “Emotional Depression”[Title/Abstract]
#3	“Meta-Analysis [Publication Type]”[Mesh] OR “Meta-Analysis as Topic”[Mesh] OR “Systematic Review”[Title/Abstract] OR “Systematic”[Title/Abstract] OR “Meta analysis”[Title/Abstract]
#4	#1 AND #2 AND #3
#5	“Cognitive Behavioral Therapy ”[Mesh] OR “Behavioral Therapy, Cognitive”[Title/Abstract] OR “Cognitive Psychotherapy”[Title/Abstract] OR “Therapy, Cognitive”[Title/Abstract]
#6	“Exercise Therapy ”[Mesh] OR “Remedial Exercise”[Title/Abstract] OR “Rehabilitation Exercise”[Title/Abstract]
#7	“Acupuncture Therapy ”[Mesh] OR “Acupuncture”[Mesh] OR “Acupressure”[Mesh] OR “Electroacupuncture”[Title/Abstract]
#8	“electric stimulation therapy”[Mesh] OR “Therapeutic Electrical Stimulation”[Title/Abstract] OR “Electrotherapy”[Title/Abstract] OR “Interferential Current Electrotherapy”[Title/Abstract]
#9	“Relaxation Therapy ”[Mesh] OR “Relaxation Technique”[Title/Abstract] OR “Nature Therapy”[Title/Abstract] OR “Ecotherapy”[Title/Abstract]
#10	“Psychotherapy”[Mesh] OR “Mindfulness”[Mesh] OR “Hyperbaric Oxygenation ”[Mesh] OR “Yoga”[Mesh] OR “Music Therapy”[Mesh] OR “Tuina ”[Title/Abstract] OR “Tai Chi ”[Title/Abstract] OR “Baduanjin”[Title/Abstract]
#11	#5 OR #6 OR #7 OR #8 OR #9 OR #10
#12	#4 AND #11

### 2.3 Types of participants

We will consider including patients definitively diagnosed with PSD (including hemorrhagic and ischemic stroke), regardless of gender, age, race, or disease duration.

### 2.4 Types of interventions

We plan to include systematic reviews or meta-analyses that use non-pharmacological therapies as experimental interventions, including non-pharmacological therapy alone or as a complementary alternative to pharmacological therapy. Non-pharmacological therapies will include psychotherapy, mindfulness intervention, hyperbaric oxygen therapy, massage, cognitive behavioral therapy, exercise therapy, yoga, acupuncture and moxibustion, electric stimulation therapy, music therapy, baduanjin exercise, tai chi, and relaxation training, and does not include the use of drugs alone. The control group received conventional treatment or a placebo. SSRIs, SNRIs, TCAs, and other medications are classified as pharmacological treatments.^[19]^

### 2.5 Types of outcomes

We will include the following outcome indicators in systematic reviews or meta-analyses. The primary outcomes will include the Hamilton Depression Rating Scale (HAMD) and Self-rating Depression Scale (SDS); The secondary outcomes will include the National Institute of Health Stroke Scale (NIHSS), Barthel Index (BI), Activities of Daily Living Scale (ADL), Minimum Mental State Examination (MMSE), and any other recognized scoring scale can be linearly converted into a standardized 100-point scale.

### 2.6 Selection of studies

**Figure 1:**
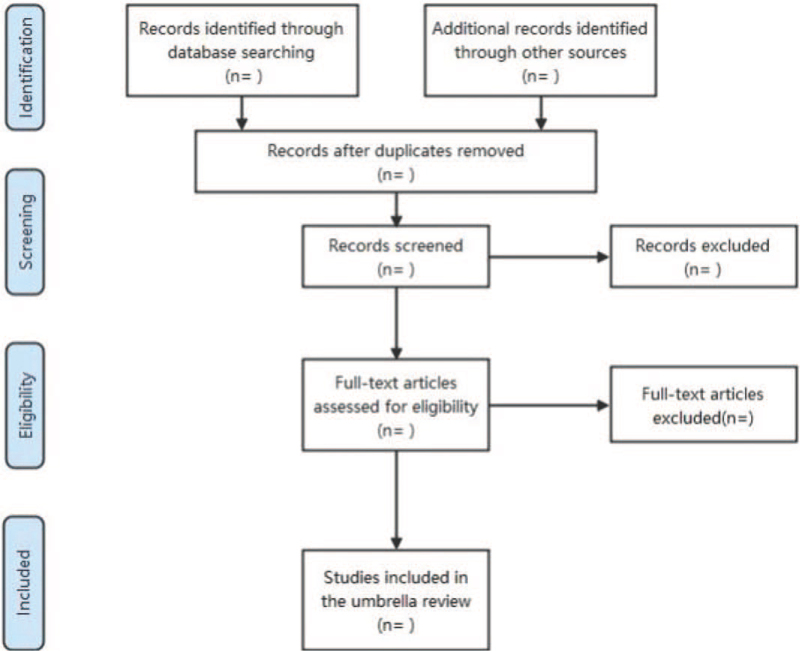
Flow diagram of study selection process.

Two reviewers (ZDL and HJQ) will conduct independent screening, referring to the established search strategy and inclusion and exclusion criteria. Reviewers first independently screened the titles and abstracts of all selected articles, excluding articles outside the scope of the predefined criteria. If necessary, we will read the full text of the downloaded articles. During the article screening process, if there is any disagreement, we invite a third reviewer for arbitration. To ensure the quality of the search, we will record the reasons for the excluded studies. If there is unavailable information, we will contact the authors of the published studies to acquire more detailed information. We did not limit the subtypes of stroke to ensure the universality of the results. Figure 1 shows the study selection process.

### 2.7 Data extraction

Data were extracted independently by 2 researchers after reading all the included studies. To extract the content included general information from the meta-analyses (first author, publication date, country, type of intervention, number of studies, total sample size), disease condition (diagnosis of PSD), research methods (name of intervention and control group, sample size per group, a specific description of the intervention, intervention frequency, and duration of treatment), and outcomes [primary and secondary outcomes, number of participants assessed, effect size (ES), and 95% confidence interval (95% CI)]. If the data are only presented in the form of graphs, we will obtain ES and its 95% CI through Ycasd29.^[20]^ If information is missing or ambiguous, we will send an email to the author of the review for accurate information. We will analyze the information collected using Epi Info (V.7.2). The integrity and accuracy of the extracted data will be verified by a third researcher.

### 2.8 Methodological quality evaluation

Two reviewers (HJQ and XQW) will use a critical assessment tool for meta-analyses (AMSTAR 2) to evaluate the quality of the included studies. AMSTAR 2 is based on the original AMSTAR, combined with multiple opinions, and formed after a strict revision procedure. AMSTAR 2 has 16 items, of which 7 domains are crucial for evaluating the validity of a review, and the evaluation of each item will be divided into “Yes,” “Partial Yes,” and “No.” We do not consider scoring the included reviews but will evaluate their quality to judge the strength of the evidence. The quality of the included reviews was divided into 4 levels: high, moderate, low, and critically low.^[21]^

### 2.9 Data analysis

We will summarize the general characteristics of qualified systematic reviews, including the total sample size, intervention measures, ES, and 95% CI. First, we will use either the fixed-effects model or random-effects model in the META package (R3.5.0) to estimate the ES and 95% CI of the eligible systematic reviews, and compare the consistency between the results we tested and the results of published reviews. Second, we will estimate the 95% prediction interval (PI) of each systematic review, and determine whether the null value is excluded.^[22]^ Next, we will explain the heterogeneity between reviews. Cochrane's Q test and *I*^2^ statistics were used to assess the heterogeneity of each systematic review. Cochrane's Q test helps judge the between-study heterogeneity; when *P* < .05, it is considered to have significant heterogeneity. *I*^2^ statistics were used to quantify the degree of heterogeneity: low, moderate, large, and very large. If *I*^2^ > 50%, this indicates substantial heterogeneity. If *I*^2^ > 75%, this indicates a very large heterogeneity.

Egger test was used to determine publication bias when *P* < .1, which indicates statistical significance. To evaluate excess significance bias, we will test whether the number of significant results O (positive results with *P* < .05) in a meta-analysis is greater than the expected number E. E is estimated from the sum of the actual power of the original studies in the meta-analysis. The O/E ratio will also be calculated to assess the severity of the bias. In addition, we will adopt the Chi-square test to evaluate the degree of bias. We believe that there is an excessively significant bias when *P* < .05. We will use the R language (version 3.5.0) to conduct statistical analysis on the basis of the data obtained.

The validity of the evidence in the meta-analysis was classified according to the corresponding criteria.^[23]^ We will use the following criteria to assess the credibility of reviews:

(1) *P* < .05 in a fixed-effects model or *P* < .001 in a random-effects model;
(2) the total number of samples in a single systematic review or meta-analysis exceed 1000;
(3) the heterogeneity between studies was small to moderate (*I*^2^ < 50%);
(4) 95% of PI values do not include null values; and
(5) no evidence of small-study effects or excess significance bias.

Evidence that fulfilled criteria (1) to (5) should be considered the strongest evidence; evidence that fulfilled criteria (1) to (4) should be considered as highly suggestive evidence; those evidence meeting criteria (1) and (2) are suggestive evidence, while the evidence that only meets criteria (1) are considered weak evidence.

## 3 Discussion

Umbrella review, also known as a review of systematic reviews, is a method of quantitatively integrating all reviews of a certain research topic, so as to draw more reliable conclusions.^[24]^ Different from narrative systematic review, umbrella reviews will recalculate the ES and assess the credibility of the evidence. Credibility is divided into 4 levels, which are divided by evaluating significant bias and small study effects. Systematic reviews have emerged to test the effectiveness of non-pharmacological treatment of PSD, but the reliability of these studies and evidence credibility need to be further explored. To the best of our knowledge, this umbrella review will be the first comprehensive assessment of current non-pharmacological treatments for PSD using quantitative methods. This review will assess the reliability of the results, and we believe that the research results will provide credible evidence for clinicians, nurses, and patients, so that effective treatment methods can be better used in clinics.

## Author contributions

The study was conceived by CCZ and LYH; ZYJ provided guidance; ZDL, HJQ, and XQW developed study inclusion criteria, research strategies, methodological quality assessment, data extraction, and statistical analysis; Manuscript writing is done by CCZ, and all members have read and approved the manuscript.

**Conceptualization:** Xiaoqian Wang.

**Data curation:** Zhendong Li, Hangjian Qiu, Xiaoqian Wang.

**Funding acquisition:** Yuejuan Zhang.

**Investigation:** Hangjian Qiu.

**Methodology:** Chengcheng Zhang, Lianyi He, Yuejuan Zhang.

**Project administration:** Zhendong Li, Hangjian Qiu, Xiaoqian Wang.

**Resources:** Yuejuan Zhang.

**Software:** Zhendong Li, Hangjian Qiu, Xiaoqian Wang.

**Supervision:** Yuejuan Zhang.

**Writing – original draft:** Chengcheng Zhang.

**Writing – review & editing:** Hangjian Qiu, Yuejuan Zhang.
